# First documented evidence of dengue and malaria co-infection in children attending two health centers in Yaoundé, Cameroon

**DOI:** 10.11604/pamj.2018.29.227.15316

**Published:** 2018-04-25

**Authors:** Gwladys Chavely Monamele, Maurice Demanou

**Affiliations:** 1 Centre Pasteur Cameroon, Yaoundé, Cameroon

**Keywords:** Dengue virus, malaria, co-infection, Cameroon

## Abstract

To date, only a few cases of malaria and dengue co-infections have been reported around the world. We describe for the first time in Cameroon, concurrent infections among children (2 to 10 years) in two health centers of Yaoundé. The two dengue strains isolated in Cameroon clustered with the Asian II genotype. Although acute concurrent infections were benign, special attention should be given to malaria and dengue co-infection in order to prevent possible severe cases.

## To the editors of the Pan African Medical Journal

Malaria and dengue are the most prevalent vector-borne diseases worldwide and represent major public health problems. These diseases present several similar signs and symptoms but dual infections were only described for the first time in 2005 [1]. To date, only a few cases of co-infections have been reported around the world. However, their clinical picture could be more severe than single infections [2]. Malaria is endemic in Cameroon but cases of dengue infections are scarcely reported [3] meanwhile there has been no documented description of Malaria and DENV co-infection. In this report, we present the results of a cross-sectional study conducted in 2012/2013 in seven health centers (HC) in three regions of Cameroon. Four HC were selected in Yaoundé (Centre region), one in Douala (Littoral region) and one in Bafoussam and Dschang (West region). Patients consulting health facilities and presenting acute fever with at least two of the nonspecific signs and symptoms such as: headache, malaise, nausea/vomiting, abdominal pain, back pain, retro-orbital pain, myalgia and arthralgia were included. The diagnosis of malaria was established by rapid diagnostic test (RDT). Venous blood was then collected into ethylenediaminetetraacetic acid (EDTA) tubes and centrifuged. Plasma was harvested, transported to the Arbovirus Laboratory of Centre Pasteur Cameroon where they were stored at -80°C until use. RDT positive samples were tested with U2 assay (UFI Panel v 2.0) developed by Waggoner et al in 2014 [4] which detects all four dengue virus (DENV) serotypes, all Leptospirosis species, Chikungunya virus and the five Plasmodium species that infect humans. PCR (Polymerase Chain Reaction) amplicons were sequenced by NGS (next-generation sequencing) on the Illumina MiSeq.

**Figure 1:**
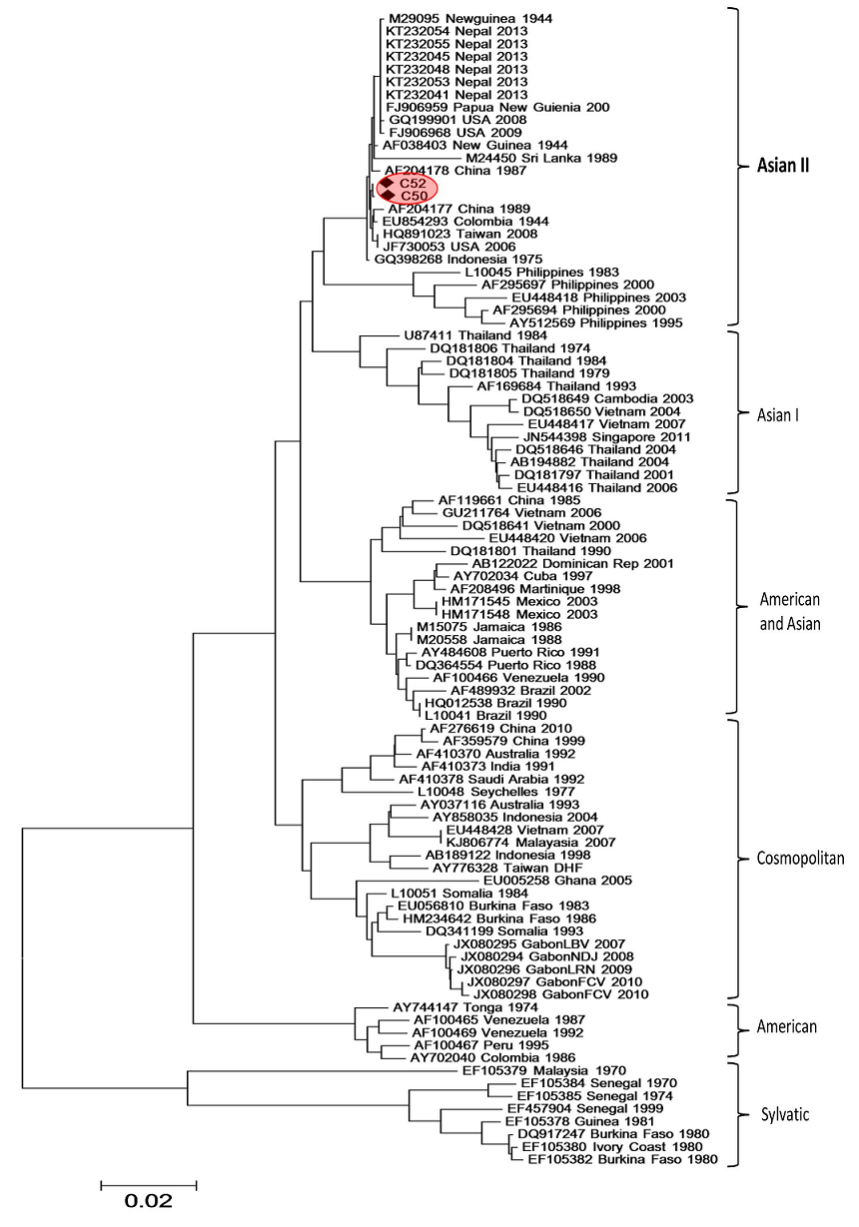
Phylogenetic tree of dengue viruses strains based on the 1376 nucleotides corresponding to partial sequences of E gene. The tree has two sequences from this study (C50 and C52)

Among the 33 RDT positive specimens tested by U2 assay, 24 (72.7%) were positive for Plasmodium among which three (12.5%) were also positive for DENV serotype 2. The three Plasmodium and DENV co-infected patients aged 2 to 10 years were all from two health facilities of Yaoundé (one patient from CASS Nkolndongo and two from CMA de Nkomo) and none was severe. They were considered clinically as malaria and antimalarial drugs were administered promptly in every case. Sequences were obtained from two of the three detected DENV (C50 and C52). Phylogenetic analyses of the Cameroon strains with other epidemiologically relevant strains showed that Cameroon strains belonged to the Asian II genotype represented by the reference strain, AF038403 New Guinea 1944 (Figure 1). This result is contradictory to the genotypes observed from other African countries that formed a distinct cluster within the Cosmopolitan genotype. This is to the best of our knowledge, the first documented description of Malaria and DENV co-infection in Cameroon. The concurrent Dengue and malaria infections prevalence observed was greater than the 2.84% and 1.6% recorded among febrile patients respectively in Nigeria [5] and in French Guiana [6]. We observed dengue infections in an unlikely population (confirmed malaria cases) which is probably the tip of the proverbial iceberg for local dengue fever epidemiology. Our study sample is precisely the opposite population of febrile illness patients who we would normally screen for active dengue infection, because we would assume that laboratory-confirmed malaria patients have been properly diagnosed. Given that the local dengue fever burden probably remains obscured by misdiagnosed malaria cases, the prevalence of DENV (and potentially, other flaviviruses) in “malaria-positive” febrile illness patients is likely to be higher.

## Conclusion

In conclusion, although acute concurrent infections were benign in this survey, special attention should be given to malaria and dengue co-infection in order to prevent possible severe cases. Since there are few documented reports on DENV genotype from malaria and DENV co-infected subjects, more strains are required in order to ascertain the role of Asian II genotype in reducing the expected severity caused by the presence of the two concomitant infections.

## Competing interests

The authors declare no competing interests.
